# The Biochemical Toxin Arsenal from Ant Venoms

**DOI:** 10.3390/toxins8010030

**Published:** 2016-01-20

**Authors:** Axel Touchard, Samira R. Aili, Eduardo Gonçalves Paterson Fox, Pierre Escoubas, Jérôme Orivel, Graham M. Nicholson, Alain Dejean

**Affiliations:** 1 CNRS, UMR Écologie des Forêts de Guyane (AgroParisTech, CIRAD, CNRS, INRA, Université de Guyane, Université des Antilles), Campus Agronomique, BP 316, Kourou Cedex 97379, France; Jerome.Orivel@ecofog.gf (J.O.); alain.dejean@wanadoo.fr (A.D.); 2 BTSB (Biochimie et Toxicologie des Substances Bioactives) Université de Champollion, Place de Verdun, Albi 81012, France; 3 Neurotoxin Research Group, School of Medical & Molecular Biosciences, University of Technology Sydney, Broadway, Sydney, NSW 2007, Australia; samira.aili@uts.edu.au (S.R.A.); graham.nicholson@uts.edu.au (G.M.N.); 4 Red Imported Fire Ant Research Center, South China Agricultural University, Guangzhou 510642, China; ofoxofox@gmail.com; 5 VenomeTech, 473 Route des Dolines—Villa 3, Valbonne 06560, France; escoubas@venometech.com; 6 Laboratoire Écologie Fonctionnelle et Environnement, 118 Route de Narbonne, Toulouse 31062, France

**Keywords:** ant venom, toxins, venom biochemistry, alkaloids, formic acid, peptides, enzymes

## Abstract

Ants (Formicidae) represent a taxonomically diverse group of hymenopterans with over 13,000 extant species, the majority of which inject or spray secretions from a venom gland. The evolutionary success of ants is mostly due to their unique eusociality that has permitted them to develop complex collaborative strategies, partly involving their venom secretions, to defend their nest against predators, microbial pathogens, ant competitors, and to hunt prey. Activities of ant venom include paralytic, cytolytic, haemolytic, allergenic, pro-inflammatory, insecticidal, antimicrobial, and pain-producing pharmacologic activities, while non-toxic functions include roles in chemical communication involving trail and sex pheromones, deterrents, and aggregators. While these diverse activities in ant venoms have until now been largely understudied due to the small venom yield from ants, modern analytical and venomic techniques are beginning to reveal the diversity of toxin structure and function. As such, ant venoms are distinct from other venomous animals, not only rich in linear, dimeric and disulfide-bonded peptides and bioactive proteins, but also other volatile and non-volatile compounds such as alkaloids and hydrocarbons. The present review details the unique structures and pharmacologies of known ant venom proteinaceous and alkaloidal toxins and their potential as a source of novel bioinsecticides and therapeutic agents.

## 1. Introduction

Nature contains a vast diversity of bioactive molecules and is hence a source of inspiration for chemists, biochemists and the pharmaceutical industry searching for molecules of potential therapeutic benefit or insecticidal activity. Amongst natural products, venoms are a promising source for the discovery of unique molecules as they offer a formidable array of biological properties. Venoms are complex cocktails of toxins that have been fine-tuned and pre-optimized during the course of evolution for greater efficacy and target selectivity towards prey capture as well as defence against predators [1]. Amongst venomous animals, insects represent a diverse group of organisms, with 120,000 extant hymenopteran species [2].

**Figure 1:**
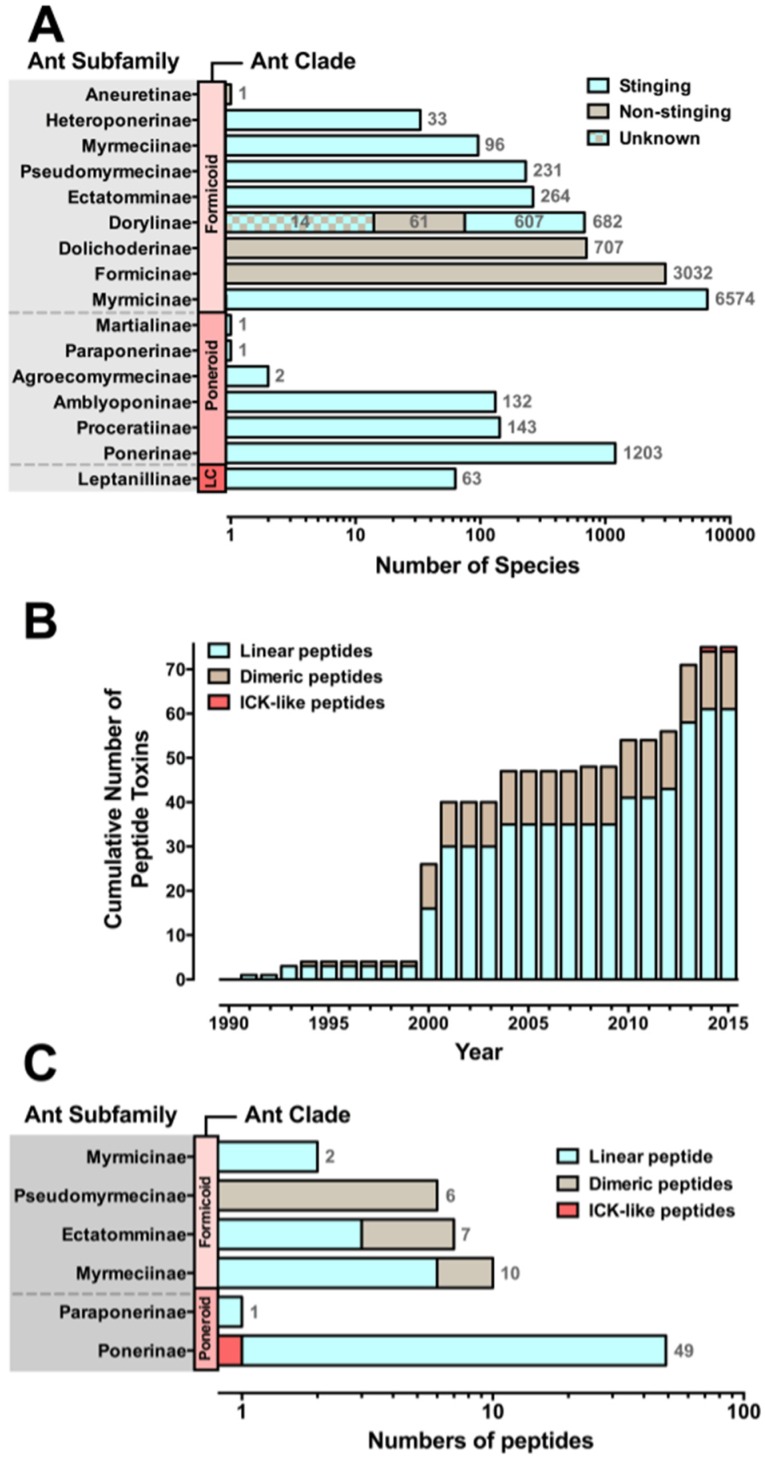
Species diversity of ant venom peptides. Panels A and B show data updated from Aili *et al.* 2014 [29]. (**A**) Ants have been grouped according to three clades, where LC represents the single-genus Leptanilloid clade. Stinging ants are represented by cyan bars and comprise around 71% of all ant species. Non-stinging ant subfamilies are depicted by brown bars. The total number of species in each subfamily is noted at the right of each bar. The newly described subfamily Dorylinae [7] is composed of several junior synonyms including stinging ants that belong to the subfamilies Aenictinae, Cerapachyinae, Ecitoninae, Leptanilloidinae, and ants belonging to the original subfamily Dorylinae that lost their ability to sting during evolution. Also, it is unclear whether the ants that once belonged to the junior synonym subfamily Aenictogitoninae, now subsumed in Dorylinae, are venomous or not, as only males have been observed and females are yet to be described [30]; (**B**) Cumulative total number of peptide-toxin sequences, showing the three main structural classes: cyan, linear peptides; brown, dimeric peptides; red, ICK-like peptides. Ant venom peptides remain barely investigated, with only 75 peptides sequenced to date; (**C**) Distribution of venom peptide structural classes by ant subfamily.

Similar to other hymenopterans, ants (Vespoidea: Formicidae) have evolved a venom apparatus that is derived from the ancestral reproductive system [3]. Ants are one of the most abundant groups of venomous organisms and dominate most terrestrial environments [4,5], with around 13,165 extant species described thus far [6] and an estimated total of ~25,000 species belonging to 16 different subfamilies [7,8,9] (Figure 1A).

Their stunning ecological diversity has also contributed to broad species diversity and, presumably, to the evolution of multiple venom types. For example, ground-dwelling ants of the subfamilies Ponerinae and Myrmicinae contain venoms that are either used for generalist predation or are specialized to prey on a locally abundant arthropod taxon (*i.e.*, isopods, myriapods, collembolans or termites). Ants that are restricted to one prey taxon and therefore may possess a specialist venom include *Psalidomyrmex procerus* (Ponerinae), which preys only on earthworms, *Strumigenys* spp. (Myrmicinae) which specializes in collembolan predation and *Megaponera analis* (Ponerinae), which preys upon a limited number of termite species [10]. While many ants overcome their prey by attacking in large numbers, some ant species are solitary hunters, suggesting that their venom is potent enough to rapidly subdue their prey similarly to solitary wasps that specifically prey on caterpillars, crickets or spiders [11].

This great taxonomical and ecological diversity has therefore allowed ants to employ their venom for several purposes such as predation and defence against predators and competitors. It can also be used for defence against microbial pathogens, communication, and as a herbicide [12,13,14]. Ant venoms have been found to contain an extraordinary diversity of toxins and other types of molecules including salts, sugars, formic acid, biogenic amines, alkaloids, free amino acids, hydrocarbons, peptides and proteins [13,15,16,17,18,19,20,21]. However, due to the small size of these organisms, the amount of venom produced by each ant is scarce in that some ants only produce around 10 μg or less of dry venom whilst other ants are capable of producing up to 300 µg, compared to spiders, scorpions and snakes that produce 0.1–300 mg of dry venom per individual [22]. This has certainly contributed to the low number of studies of ant venoms. Nevertheless, the present review aims to describe the current knowledge of the wide range of toxins present in ant venoms and their functional roles.

## 2. Toxins from Non-Stinging Ants

Of all ant species, only 71% are considered to be stinging species due to the fact that a few ant subfamilies have lost their ability to sting over the course of evolution. Instead of injecting their venoms, Formicinae spray their venom (which is secreted by the venom gland), whereas Dolichoderinae and Aneuretinae spray their targets with substances (*i.e.*, ketones and iridoïds) secreted by their pygidial glands [23]. Among the army ants (Dorylinae), ants from the genus *Dorylus* have a non-functional stinger and their venom glands are a source of trail pheromones. *Dorylus* species do not employ venom for prey immobilization but overcome their prey as a result of their overwhelming numbers (group hunting behaviour) and their disabling bites (Figure 1A) [24].

While stinging species inject their secretions with a stinger, stingless ants from the subfamily Formicinae spray their venoms through a special opening called the acidopore, a round orifice surrounded by a fringe of hairs constituting a unique feature of formicine ants. The peculiarity of this venom apparatus has considerably affected the chemical nature of the components secreted by the venom glands, promoting the natural selection of volatile compounds. Formic acid (methanoic acid) is the predominant compound and is presumably present in the venoms of all Formicinae, although acetic acid can also be present [25]. Formic acid, present in concentrations of up to 70% (*v*/*v*), is an alarm pheromone that, along with acetic acid, is an efficient defensive compound against competitors and predators, including vertebrates [23,26]. The major precursors for its biosynthesis are the amino acids serine and glycine [27]. By self-grooming their acidopore, *Lasius neglectus* (Formicinae) workers uptake venom into their mouth and apply the acid on brood in their colony in order to inhibit the growth of fungal pathogens [25]. Also, upon occasional aggressive encounters, *Nylanderia fulva* (Formicinae) workers can apply their formic acid-rich venom onto their cuticle to detoxify venom alkaloids of the fire ant *Solenopsis invicta* (Myrmicinae) [28].

Several arboreal ant species use their venom gland secretion as a herbicide to destroy plants, mainly encroaching vines that compete with their host plant. An example of this is the Amazonian species *Myrmelachista schumanni* (Formicinae) that creates the “devil′s gardens”—large stands of trees almost exclusively comprised of *Duroia hirsuta*, a myrmecophyte sheltering *M. schumanni* colonies in its hollow stems (domatia). Workers of this plant-ant species kill all trees situated around their host plants with their venom, which is mostly composed of formic acid. This facilitates the growth and establishment of their host plant [14,31].

## 3. Peptides

### 3.1. Ant Venom Peptides

In most animal venoms, peptides (<100 amino acids) are the predominant class of toxins and have been investigated extensively in organisms such as scorpions, cone snails, and spiders [32,33,34,35]. Although ant venoms remain very much unexplored, recent studies have revealed that the venoms of stinging ants (those belonging to the subfamilies Paraponerinae, Ponerinae, Amblyoponerinae, Dorylinae, Myrmeciinae, Pseudomyrmecinae, Myrmicinae, and Ectatomminae) are also rich in peptides, similar to other venomous animals [36,37]. To date, 75 venom peptides from only six ant subfamilies (11 ant species) have been fully sequenced (Figure 1B) [29]. These peptides have previously been classified based on their structure into three main groups: linear, dimeric, and inhibitor cystine knot (ICK)-like peptides (for a complete review of ant venom peptides, see Aili *et al.* [29]). An alternate approach to classifying ant venom peptides is to base the nomenclature on biological activity: cytolytic and neurotoxic peptides. This review will address these ant venom peptides based on their biological functions.

#### 3.1.1. Cytolytic Peptides

Most proteomic studies on ant venoms have confirmed the prevalence of small, linear peptides (devoid of disulfide bonds) with fewer than 35 residues [19,36,37]. Most of these small peptides are cytolytic and often possess insecticidal, haemolytic and/or antimicrobial properties. Examples include ponericins from the ant *Neoponera goeldii* (Ponerinae; formerly *Pachycondyla goeldii*) that exhibit haemolytic activity, antibacterial activity against both Gram-positive and Gram-negative bacteria, as well as insecticidal activity [12]. Ponericins have been classified into three different families (“G”, “W”, and ”L”) based on sequence homology [29]. Numerous additional ponericin-like peptides were also sequenced from the venom of *Neoponera apicalis* and *N. inversa* (Ponerinae; formerly *Pachycondyla apicalis* and *P. inversa*) [38]. Thus, although the biological function of these peptides has not been characterized, their strong homology to G-, W- and L-family ponericins from *N. goeldii* suggests that they should have both antimicrobial and insecticidal activities, however, this remains to be proven. Additional homologous toxins include dinoponeratoxins from both *Dinoponera australis* and *D. quadriceps* (Ponerinae) [37,39], and bicarinalins from *Tetramorium bicarinatum* (Myrmicinae) [40]. Multiple alignment analyses have shown that these linear venom peptides display various degrees of sequence homology to each other and that they can be separated into several families [41,42,43,44]. More recently, three novel antimicrobial linear peptides with little homology to ponericin peptides have been isolated from the venom of the ant *Ectatomma brunneum* (Ectatomminae; formerly known as *E. quadridens*) [45].

Another group of peptides from ant venoms are pilosulins that constitute the major allergens of the venom of *Myrmecia pilosula* (Myrmeciinae). Pilosulin 1 is a long linear peptide (57 amino acids) and displays haemolytic and cytolytic activities [46,47]. Pilosulins 3, 4, and 5 are a group of homo- and heterodimeric peptides. Although these peptides possess some antimicrobial activity, and are classified as allergens, their biological function remains unknown [41,42].

The biological function of such membrane perturbing peptides in ant venoms is likely to be varied. Among spider and scorpion venoms, cytolytic peptides are believed to act as membrane-disrupting agents, facilitating the passage of other disulfide-rich neurotoxins through cellular barriers to their molecular targets [48]. These linear cytolytic peptides also have direct toxic effects on prey, although this insecticidal activity is often moderate in comparison to disulfide-rich neurotoxins [32]. However, in some cases, spiders and scorpions use cytolytic-based venoms rather than neurotoxic-based venoms [49,50,51]. For example, the cyto-insectotoxins present in the venom of the spider *Lachesana tarabaevi* have a potent insecticidal effect and are the major insecticidal toxins in this venom [52]. Most ants seem to have evolved cytolytic-based venoms [12,36,37] and have probably developed a strategy similar to that of the spider *L. tarabaevi* for subduing their prey. The cytolytic peptides seen in most ant venoms act synergistically against different kinds of cells, disrupting membranes and rapidly killing prey.

Due to their non-selective activity, membrane-perturbing venom peptides are able to target the membranes of bacterial cells and, therefore, often exhibit some antimicrobial activity. This antimicrobial activity may be a bonus function for ants as it helps with the social immunity of the colony [53,54]. In eusocial insects, promiscuity and the relative genetic homogeneity of individuals creates ideal circumstances for the spread of infectious diseases in their nests. Therefore, the presence of multiple membrane-perturbing peptides with antimicrobial activities in ant venoms is also believed to be a way to disinfect captured prey before to bringing them back to the nest [12]. Another hypothetical function would be to assist in pre-digestion of prey. This is important since adult ants only feed on liquids due to their inability to digest solid food as a result of their narrow and constricted waists. Cytolytic activity combined with an enzymatic activity would help the degradation of cellular membranes of prey and, therefore, liquefy prey as do spider venoms [55].

Membrane-perturbing peptides are promising candidates for the future development of novel antimicrobial, insecticidal, and anticancer drugs, and have been well investigated for this purpose for many years. However, pharmaceutical research has struggled to find valid lead drug candidates as, to date, these peptides cannot sufficiently discriminate between the membranes of pathogenic cells or erythrocytes and other human host cells [48].

#### 3.1.2. Neurotoxic Peptides

Neurotoxic peptides are widely expressed in animal venoms to assist in the rapid immobilization of prey. These neurotoxins act on a broad diversity of molecular targets, mostly ion channels, with varied selectivity, specificity, and efficacy. Many peptidic toxins modulating ion channels have been discovered in arthropod venoms. As several ant venoms have paralytic effects on arthropods, it is clear that they also contain neurotoxins that induce paralysis [11,56]. However, studies investigating the neurotoxic properties of ant venom peptides are rare and only two neurotoxic peptides have been characterized so far, as discussed below.

The first neurotoxic peptide that was isolated and characterized was poneratoxin, a small 25 residue linear peptide derived from the bullet ant *Paraponera clavata* (Paraponerinae). It has been shown to be capable of modulating voltage-gated sodium (Na_v_) channels of both vertebrates and invertebrates, blocking synaptic transmission in the insect CNS [57,58]. Poneratoxin causes repetitive firing and prolongation of action potentials due to the presence of a slowly developing inward sodium current that activates at hyperpolarizing potentials. This results from a potential toxin-induced interconversion between a fast and a slow conducting state of the Na_V_ channel [59,60,61]. Due to its high efficacy, this peptide has been used in the construction of a novel bioinsecticide employing a recombinant poneratoxin-producing baculovirus [61].

The other neurotoxic peptide isolated from ant venom is a dimeric peptide, Ectatomin Et-1, from the ant *Ectatomma tuberculatum* which has been found to be the most potent neurotoxic peptide isolated from ant venoms [62]. This peptide, which is one of a family of four related peptides from *Ectatomma* spp., is a voltage-gated calcium (Ca_v_) channel blocker, and also a pore-forming peptide cytotoxic to vertebrate and invertebrate cells [63,64].

#### 3.1.3. Uncharacterized Peptides

Dimeric peptides are highly stable as they comprise two subunits that are linked by one or several disulfide bonds. Among ant venoms, dimeric peptides seem to be common in the formicoid subfamilies Ectatomminae, Pseudomyrmecinae, and Myrmeciinae (Figure 1C). In addition to pilosulins and ectatomins, the myrmexins are a group of six heterodimeric peptides isolated from the venom of *Pseudomyrmex triplarinus* (Pseudomyrmecinae) whose biological functions remain unknown [65]. Homo- and heterodimeric peptides have also been shown to be present in the venoms of *P. penetrator* [66] and *Tetraponera* sp. (Pseudomyrmecinae) [36] although their amino acid sequences and biological activity also remain uncharacterized.

The recent transcriptome analysis of the venom glands of the giant ant *Dinoponera quadriceps* has confirmed the sequence of a third structural class of ant venom peptides: the ICK-like peptides. ICK peptides contain three disulfide bonds, forming a pseudo knot, and are very stable. These are present in the venoms of cone snails and spiders, and typically have neurotoxic properties [67,68]. These peptides are a minor component of the venom of the giant ant, and their role and biological activity is still unknown [69].

Until recently, the limited amount of venom has restricted the biochemical characterization of ant venom peptides. However, recent investigations using high resolution technologies to probe ant venom peptidomes have revealed the vast and unexplored structural diversity of peptidic toxins with many small, linear peptides as well as several peptides structured by disulfide bonds that constitute novel structural classes of toxins with a likely novel, associated pharmacology [19,36,66]. Unfortunately, the number of characterized ant venom peptides is vanishingly small compared with the enormous peptide diversity revealed among ant venoms. This diversity, combined with the great ecological and taxonomical diversity of ants, suggests that ant venom peptides constitute a promising new source in the search for both novel drugs and insecticides.

### 3.2. Proposed Rational Nomenclature System for Ant Venom Peptides

Cutting-edge technologies such as integrated venomics represent a new gateway to exploring the venom peptides of small organisms such as ants [70,71,72], and have led to increases in the rate of description of novel peptidic toxins. Stinging ant venoms represent an untapped source of toxins, and the total number of peptides has been estimated to be in excess of 1 million [36]. Thus far, there is no consistent nomenclature for naming newly identified peptidic toxins in ant venoms. This may cause considerable confusion, as presumably at some point there will be a surge of ant venom-derived toxins being identified with the advent of new, more rapid and sensitive analytical strategies. It will also be difficult to quickly compare toxins and establish evolutionary relationships with no consistent nomenclature. The use of the same toxin name for different peptidic toxins with similar functions regardless of the relatedness of the source ant species is advantageous, as it allows for the quick identification of the peptide′s properties; however, it does not reveal any evolutionary relationships. This is illustrated by the ponericins, a family of antimicrobial peptides, which were originally isolated from three different species in the genus *Neoponera* (Ponerinae) [12,38] and later from the unrelated ant *Ectatomma brunneum* (Ectatomminae) [45]. Another issue is the use of multiple names for the same toxin, such as with Myr p 1, pilosulin 1, and Myr p 1.0101, where all names refer to the same peptide, a linear allergenic peptide from the venom of *Myrmecia pilosula* [47,73]. Table 1 also highlights the confusing similarity in the names of ant venom alkaloids *vs.* peptides (e.g., solenopsins *vs.* ponericins) where toxins have completely different biochemical structure and function yet they are simply named after the organism from which they were obtained.

**Table 1:** Generic names for peptidic toxins from stinging ant subfamilies and their corresponding abbreviations.

Subfamily	Generic Toxin Name	Toxin Abbreviation
Agroecomyrmecinae	Agroecomyrmecitoxin	AGRTX
Amblyoponerinae	Amblyotoxin	ABTX
Dorylinae	Dorylitoxin	DRTX
Ectatomminae	Ectatotoxin	ECTX
Heteroponerinae	Heteroponeritoxin	HETX
Leptanilinae	Leptanilitoxin	LETX
Martialinae	Martialitoxin	MATX
Myrmeciinae	Myrmeciitoxin	MIITX
Myrmicinae	Myrmicitoxin	MYRTX
Paraponerinae	Paraponeritoxin	PPOTX
Ponerinae	Poneritoxin	PONTX
Proceratiinae	Proceratoxin	PROTX
Pseudomyrmecinae	Pseudomyrmecitoxin	PSDTX

This highlights the unmet need for a standardized nomenclature system for ant venom peptides in order to avoid confusion. Wiese *et al.* (2004) proposed a standardized nomenclature system for the *Myrmecia pilosula* venom allergens according to the International Union of Immunological Societies (IUIS) [74]. Although this system has been very useful for the pilosulins, it does not seem very practical for the naming of all ant venom peptides. This is because it provides no details on the biological activity or the molecular target of the toxin and it provides only minimal taxonomic information with no reference to subfamilies. It is therefore of great importance to adopt a nomenclature with sufficient detail and which follows the patterns of nomenclature used for other venomous organisms.

Accordingly, we propose adopting the standard nomenclature system used in naming spider [75], centipede [76] and sea anemone venom peptides [77] for naming ant venom peptides. The nomenclature is as follows:

- The toxin name should begin with a Greek letter prefix denoting the biological activity or molecular target (if known) of the peptide; see King *et al.* for a summary [75]. Where the target is not known the toxin should have a prefix of “U”. As only a few pharmacological activities have been determined to date this will be an ongoing process. Haemolytic, cytolytic or antibacterial peptides that have activity against bacteria, fungus, insect or vertebrate cells are denoted by the Greek letter “M” to denote a general action to cause membrane perturbation. Neurotoxic peptides (*i.e.*, poneratoxin and ectatomin) which target voltage-gated sodium or calcium ion channels have been identified by the prefixes “δ” and “ω”, respectively.
- The Greek letter prefix will be followed by a generic toxin name. As all ants are grouped into a single family (Formicidae), we propose to slightly modify King′s nomenclature which uses family names and use the 13 extant stinging subfamily names instead (Figure 1A). This will allow the toxins to be compared and will highlight the evolutionary relationship between different toxins. A list of the proposed generic toxin names and their corresponding abbreviations is proposed in Table 1 for all extant subfamilies of stinging ants. These names and their abbreviations have been carefully chosen so that they do not overlap with current toxins from other venomous animals nor other chemical groups. NB: non-stinging ants are thought to contain mostly non-peptidic venom components, and are therefore not included.
- The toxin name is then followed by an uppercase letter that indicates the genus of the ant and a lowercase letter which identifies the species of the ant from which it was isolated. An additional one or two lowercase letters may be required to distinguish species with the same first letters. Due to several taxonomic revisions concerning ants, their species names are often subject to modifications; therefore, all ant venom studies should follow the world′s largest online ant database AntCat [6] when defining the most current species name.
- Finally, an alpha-numerical code will be used to separate different structural classes of peptides based on their molecular scaffold and amino acid sequences. An Arabic numeral will be used to distinguish different toxins from the same species with little amino acid homology or different three-dimensional structures. A lowercase letter will also be added in order to distinguish isotoxins. The isotoxins are named based on the sequence alignment analyses presented in the review of Aili *et al.* [29]. The definition of isotoxin groups by Olivera *et al.* [77] will be used to distinguish isotoxins. Toxins from the same ant species will be classified in the same isotoxin group when there is ≥ 50% similarity in molecular size, biological function as well as amino acid sequence.

We have applied this proposed nomenclature to all the known peptidic toxins isolated and sequenced from the venoms of both poneroid (Table 2) and formicoid (Table 3) ants. Using this rational nomenclature method, we have uniformly renamed the 75 currently sequenced peptidic ant toxins. This new nomenclature will provide a clearer means of identifying and classifying former toxins as well as new peptides, which will facilitate future exploration of ant venom peptides.

**Table 2:** Venom peptide toxins from poneroid ant species renamed according to the proposed rational nomenclature system.

Species (Subfamily)	Original Toxin Name	Proposed Toxin Name	Abbreviation	Reference
*Paraponera clavata* (Paraponerinae)	Poneratoxin	δ-Paraponeritoxin-Pc1a	δ-PPOTX-Pc1a	[57]
*Neoponera goeldii* (Ponerinae)	Ponericin G1	M-poneritoxin-Ng3a	M-PONTX-Ng3a	[12]
	Ponericin G2	U_1_-poneritoxin-Ng3b	U_1_-PONTX-Ng3b	[12]
	Ponericin G3	M-poneritoxin-Ng3c	M-PONTX-Ng3c	[12]
	Ponericin G4	M-poneritoxin-Ng3d	M-PONTX-Ng3d	[12]
	Ponericin G5	U_1_-poneritoxin-Ng3e	U_1_-PONTX-Ng3e	[12]
	Ponericin G6	M-poneritoxin-Ng3f	M-PONTX-Ng3f	[12]
	Ponericin G7	U_1_-poneritoxin-Ng3g	U_1_-PONTX-Ng3g	[12]
	Ponericin L1	U_1_-poneritoxin-Ng2a	U_1_-PONTX-Ng2a	[12]
	Ponericin L2	M-poneritoxin-Ng2b	M-PONTX-Ng2b	[12]
	Ponericin W1	M-poneritoxin-Ng1a	M-PONTX-Ng1a	[12]
	Ponericin W2	U_1_-poneritoxin-Ng1b	U_1_-PONTX-Ng1b	[12]
	Ponericin W3	M-poneritoxin-Ng1c	M-PONTX-Ng1c	[12]
	Ponericin W4	M-poneritoxin-Ng1d	M-PONTX-Ng1d	[12]
	Ponericin W5	M-poneritoxin-Ng1e	M-PONTX-Ng1e	[12]
	Ponericin W6	M-poneritoxin-Ng1f	M-PONTX-Ng1f	[12]
*Neoponera inversa* (Ponerinae)	Ponericin Pi I1	U_1_-poneritoxin-Ni3a	U_1_-PONTX-Ni3a	[38]
	Ponericin Pi I2	U_1_-poneritoxin-Ni3b	U_1_-PONTX-Ni3b	[38]
	Ponericin Pi I3	U_1_-poneritoxin-Ni3c	U_1_-PONTX-Ni3c	[38]
	Ponericin Pi I4	U_1_-poneritoxin-Ni3d	U_1_-PONTX-Ni3d	[38]
	Ponericin Pi II1	U_1_-poneritoxin-Ni1a	U_1_-PONTX-Ni1a	[38]
	Ponericin Pi II2	U_1_-poneritoxin-Ni1b	U_1_-PONTX-Ni1b	[38]
	Ponericin Pi III1	U_1_-poneritoxin-Ni2a	U_1_-PONTX-Ni2a	[38]
*Neoponera apicalis* (Ponerinae)	Ponericin Pa I1	U_1_-poneritoxin-Na3a	U_1_-PONTX-Na3a	[38]
	Ponericin Pa I2	U_1_-poneritoxin-Na3b	U_1_-PONTX-Na3b	[38]
	Ponericin Pa II 1	U_1_-poneritoxin-Na1a	U_1_-PONTX-Na1a	[38]
	Ponericin Pa II 2	U_1_-poneritoxin-Na1b	U_1_-PONTX-Na1b	[38]
	Ponericin Pa IV1	U_1_-poneritoxin-Na2a	U_1_-PONTX-Na2a	[38]
*Dinoponera australis* (Ponerinae)	Dinoponeratoxin Da-1039	U_1_-poneritoxin-Da1a	U_1_-PONTX-Da1a	[39]
	Dinoponeratoxin Da-1585	U_1_-poneritoxin-Da3a	U_1_-PONTX-Da3a	[39]
	Dinoponeratoxin Da-1837	U_1_-poneritoxin-Da2a	U_1_-PONTX-Da2a	[39]
	Dinoponeratoxin Da-2501	U_1_-poneritoxin-Da3b	U_1_-PONTX-Da3b	[39]
	Dinoponeratoxin Da-3105	U_1_-poneritoxin-Da4a	U_1_-PONTX-Da4a	[39]
	Dinoponeratoxin Da-3177	M-poneritoxin-Da4b	M-PONTX-Da4b	[39]
*Dinoponera quadriceps* (Ponerinae)	Dinoponeratoxin Dq-762	U_1_-poneritoxin-Dq1a	U_1_-PONTX-Dq1a	[37]
	Dinoponeratoxin Dq-987	U_1_-poneritoxin-Dq1b	U_1_-PONTX-Dq1b	[37]
	Dinoponeratoxin Dq-1031	U_1_-poneritoxin-Dq1c	U_1_-PONTX-Dq1c	[37]
	Dinoponeratoxin Dq-1062	U_1_-poneritoxin-Dq2a	U_1_-PONTX-Dq2a	[37]
	Dinoponeratoxin Dq-1133	U_1_-poneritoxin-Dq2b	U_1_-PONTX-Dq2b	[37]
	Dinoponeratoxin Dq-1289	U_1_-poneritoxin-Dq2c	U_1_-PONTX-Dq2c	[37]
	Dinoponeratoxin Dq-1839	U_1_-poneritoxin-Dq3a	U_1_-PONTX-Dq3a	[37]
	Dinoponeratoxin Dq-1840	U_1_-poneritoxin-Dq3b	U_1_-PONTX-Dq3b	[37]
	Dinoponeratoxin Dq-1856	U_1_-poneritoxin-Dq3c	U_1_-PONTX-Dq3c	[37]
	Dinoponeratoxin Dq-1897	U_1_-poneritoxin-Dq3d	U_1_-PONTX-Dq3d	[37]
	Dinoponeratoxin Dq-1984	U_1_-poneritoxin-Dq3e	U_1_-PONTX-Dq3e	[37]
	Dinoponeratoxin Dq-3104	M-poneritoxin-Dq4a	M-PONTX-Dq4a	[37]
	Dinoponeratoxin Dq-3162	M-poneritoxin-Dq4b	M-PONTX-Dq4b	[37]
	Dinoponeratoxin Dq-3163	U_1_-poneritoxin-Dq4c	U_1_-PONTX-Dq4c	[37]
	Dinoponeratoxin Dq-3178	U_1_-poneritoxin-Dq4d	U_1_-PONTX-Dq4d	[37]
	Dinoponeratoxin ICK-like	U_1_-poneritoxin-Dq5a	U_1_-PONTX-Dq5a	[69]

**Table 3:** Venom peptide toxins from formicoid ant species renamed according to the proposed rational nomenclature system.

Species (Subfamily)	Original Toxin Name	Proposed Toxin Name	Abbreviation	Reference
*Tetramorium bicarinatum* (Myrmicinae)	Bicarinalin 1	M-myrmicitoxin-Tb1a	M-MYRTX-Tb1a	[40]
	P 17	U_1_-myrmicitoxin-Tb2a	U_1_-MYRTX-Tb2a	[40]
*Ectatomma tuberculatum* (Ectatomminae)	Ectatomin-Et1	ω/M-ectatotoxin-Et1a	ω/M-ECTX-Et1a	[62]
	Ectatomin-Et2	U_1_-ectatotoxin-Et1b	U_1_-ECTX-Et1b	[78]
*Ectatomma brunneum* (Ectatomminae)	Ectatomin-Eq1	U_1_-ectatotoxin-Eb1a	U_1_-ECTX-Eb1a	[78]
	Ectatomin-Eq2	U_1_-ectatotoxin-Eb1b	U_1_-ECTX-Eb1b	[78]
	Ponericin-Q42	M-ectatotoxin-Eb2a	M-ECTX-Eb2a	[45]
	Ponericin-Q49	M-ectatotoxin-Eb2b	M-ECTX-Eb2b	[45]
	Ponericin-Q50	M-ectatotoxin-Eb2c	M-ECTX-Eb2c	[45]
*Pseudomyrmex triplarinus* (Pseudomyrmecinae)	Myrmexin I	U_1_-pseudomyrmecitoxin-Pt1a	U_1_-PSDTX-Pt1a	[65]
	Myrmexin II	U_1_-pseudomyrmecitoxin-Pt1b	U_1_-PSDTX-Pt1b	[65]
	Myrmexin III	U_1_-pseudomyrmecitoxin-Pt1c	U_1_-PSDTX-Pt1c	[65]
	Myrmexin IV	U_1_-pseudomyrmecitoxin-Pt1d	U_1_-PSDTX-Pt1d	[65]
	Myrmexin V	U_1_-pseudomyrmecitoxin-Pt1e	U_1_-PSDTX-Pt1e	[65]
	Myrmexin VI	U_1_-pseudomyrmecitoxin-Pt1f	U_1_-PSDTX-Pt1f	[65]
*Myrmecia pilosula* (Myrmeciinae)	Myr p 157–112	M-myrmeciitoxin-Mp1a	M-MIITX-Mp1a	[79]
	Myr p 1 57–112 (Ile5)	M-myrmeciitoxin-Mp1b	M-MIITX-Mp1b	[79]
	Myr p 1 65–112	M-myrmeciitoxin-Mp1c	M-MIITX-Mp1c	[79]
	Myr p 1 68–112	M-myrmeciitoxin-Mp1d	M-MIITX-Mp1d	[79]
	Myr p 1 71–112	M-myrmeciitoxin-Mp1e	M-MIITX-Mp1e	[79]
	Myr p 1 86–112	U_1_-myrmeciitoxin-Mp1f	U_1_-MIITX-Mp1f	[79]
	Pilosulin 3a	M-myrmeciitoxin-Mp2a	M-MIITX-Mp2a	[41]
	Pilosulin 3b	M-myrmeciitoxin-Mp2b	M-MIITX-Mp2b	[41]
	Pilosulin 4	M-myrmeciitoxin-Mp3a	M-MIITX-Mp3a	[41]
	Pilosulin 5	M-myrmeciitoxin-Mp4a	M-MIITX-Mp4a	[42]

## 4. Ant Venom Proteins

Although ants are amongst the most abundant and diverse of all social insects [5], there remains limited information in the literature regarding their venom protein characteristics. Most published studies have investigated the allergenic properties of ant venoms [43,74,80]. This is especially true for proteomic data, even though such information can give insights into the functions of venom components [81]. This paucity of data is mainly due to the limited amount of venom that can be obtained from stinging ants [82] and the laborious nature of venom dissections and extractions [83,84]. Nevertheless, current data shows that ant venom proteins are highly diverse, as is the case with the peptide component. This diversity is further exemplified with the vastly different patterns of venom protein expression across ant subfamilies which has been attributed to both phylogenetic and behavioural differences between ants [82,85].

One of the first studies to report the presence of proteins in ant venoms was that of Leluk *et al.* [81] which found proteins ranging from 24 to 75 kDa in all six ants investigated *(Dinoponera grandis*, *Diacamma* sp., *Paraponera clavata*, *Odontoponera transversa*, *Pogonomyrmex rugosus*, and *Po. maricopa*). The two most investigated ants using proteomics are *Myrmecia pilosula* (Australian jack jumper ant) and *Solenopsis invicta* (red imported fire ant) due to frequent allergic reactions to their sting which can lead to anaphylaxis and, in some extreme cases, death [83,85]. In fact, the first ever published study proving the presence of proteins in ant venoms was performed on the red imported fire ant in 1979, where the authors managed to extract and enzymatically assay venom proteins by employing chromatographic separation on a massive amount of manually-milked venom (*ca.* 120 mg) [86].

*Myrmecia pilosula* venom is mainly composed of peptides, however, it does contain six proteins between 26 and 90 kDa [43,74]. Most of its activity was originally attributed to the pilosulin peptides (see Section 3.1.1), however, it was later found that the proteins also play a role in the allergic reactions [43,74,80,87]. An interesting feature of this venom, which had hindered investigations of its composition in the past, is its highly basic nature which makes venom proteins more difficult to separate based on isoelectric point (p*I*) when using two-dimensional polyacrylamide gel electrophoresis (2D-PAGE) [80]. The basic nature of these venom proteins is common with defensive venoms, such as that of bees, and the proteins responsible for this effect usually cause painful or cytolytic effects [81]. However, this is not common amongst ants, as the original study performed by Leluk *et al.* (1989) revealed that the majority of the six ants investigated contained acidic venoms, particularly that of *Paraponera clavata* [81].

The proteome of *Solenopsis invicta* has only recently been investigated using 2D-PAGE based on a commercial protein extract, due to the small amount of protein present in the venom in comparison to its high alkaloid content (>95% alkaloids; see Section 5.2) [74,84,88]. It has been postulated that one reason for *S. invicta* venom being less proteinaceous than those of other ant venoms is that this ant evolved more recently compared to the more ancestral ants with higher venom protein content [89]. Other venoms that have been shown to be proteinaceous in nature are those from Ponerinae, Dorylinae, Pseudomyrmecinae (e.g., *Pseudomyrmex triplarinus* with proteins making up 42% of the venom′s dry weight [90]), and even some Myrmicinae such as *Myrmica* spp. and *Pogonomyrmex* spp.

The proteins identified in ant venoms have been assigned to one of the following three major functional protein groups: housekeeping proteins, body muscle proteins, or true venom proteins (classification modified from [83]). Previous transcriptomic studies have revealed that the majority of transcripts identified from the venom glands (~40%–65%) are housekeeping proteins such as ribosomal proteins which come from the venom gland tissues [69,85,87]. These predicted housekeeping and body muscle proteins have also been predicted by other approaches such as proteomics [83] and are therefore not covered in this review as they are not true venom components. Transcriptomics has revealed that true venom proteins make up a small fraction of the transcripts being expressed in venom gland tissues (<1%–5%).While there are indications of several new venom gland components using transcriptomics, one must be cautious in considering that not all potential transcripts identified are necessarily translated into proteins, and must be confirmed using proteomic techniques. Therefore, true venom components which have been confirmed by proteomic studies have been categorized into (1) toxic venom proteins; (2) non-toxic proteins involved in protecting other venom components and the gland tissue; and (3) non-toxic proteins involved in chemical communication.

### 4.1. Toxic Venom Proteins

The present review will discuss those proteins which have been associated with venom diffusion and toxicity to prey or predators as well as major allergenic proteins revealed by venom gland proteomic and transcriptomic studies. These proteins can be further classified into five subgroups: (1) neurotoxins; (2) proteins that promote venom diffusion or modulate prey defense mechanisms; (3) proteins that promote tissue damage or cause inflammation; (4) allergens; and (5) antimicrobial proteins involved in colony/food asepsis. Ant venom toxic proteins commonly interfere with tissue signalling, lipid homeostasis, protein–protein interactions or trafficking of vesicles [69]. While only two ant venom gland transcriptomes have been published to date, these have revealed an enormous amount of novel information regarding potential proteins in the venom gland [69,85,91]. It is clear that further transcriptomic studies are necessary, as it would make the current difficult task of novel protein annotation a lot clearer [83]. Moreover, a significant number of predicted proteins are apparently unique to ant venoms, as they are not homologous to previously deposited sequences in databanks from other tissues or organisms for the first time [69,85,91].

#### 4.1.1. Neurotoxic Proteins

An increasing number of proteins that cause neurotoxicity are being revealed in ant venoms. For example, the proteomic investigation of *Solenopsis invicta* venom [83] revealed the presence of three 18–43.1 kDa neurotoxins similar to proteins from other arthropods. One of these proteins is homologous to U_5_-ctenitoxin-Pk1a-like protein which has been implicated in causing spastic paralysis in mice [92]. The other protein found is homologous to the neurotoxic alpha-toxin Tc48a-like protein, which is also lethal to mice through its action on Na_V_ channels [83,93]. The third neurotoxic protein was homologous to *Scolopendra* (Chilopoda) toxin-like proteins which are not lethal to vertebrates, but are neurotoxic to insects and crustaceans [94,95].

Phospholipases have been described as one of the major proteins in several hymenopteran venoms and are considered potent neurotoxic, cytotoxic and allergenic proteins [82,96]. Phospholipases (PL) hydrolyze the different ester linkages in phospholipids. There are five major types: PLA_1_, PLA_2_, and PLC (which cleave ester bonds at positions sn-1, sn-2, and sn-3, respectively), PLD (which is mainly found in plants that attacks the nitrogenous base of the phospholipids) and PLB (which cleaves lysophospholipids) [96]. The most commonly reported phospholipase in ant venoms is PLA_2_ [83,97,98,99], however, there have been isolated reports of PLA_1_, PLB, and PLD as well [69,99,100]. Venom phospholipases from various animals have been demonstrated to induce neurotoxicity, platelet activation, allergic reactions, haemolysis, and tissue damage. Unlike snake venom phospholipases which are lethal to their prey [69,101], ant venom phospholipases have not been implicated in causing lethality of prey, however, it is likely that they have synergistic activity with other toxic proteins which cause lethality [83,102].

#### 4.1.2. Proteins that Promote Venom Diffusion or Modulate Victim Defense Mechanisms

Examples of proteins involved with tissue damage would include phospholipases, hyaluronidases, proteases, and venom acid phosphatases. Hyaluronidase is implicated in aiding the spread of venom through the host tissues. This results from the hydrolysis of hyaluronic acid and chondroitin sulphate which are essential components of connective tissues [82], thereby increasing membrane permeability, reducing viscosity, and making tissues more permeable to venom neurotoxins [82,103]. This function has been used clinically to assist in the absorption of fluids administrated by subcutaneous or intramuscular injection, and to improve the diffusion of local anaesthetics [82]. Hyaluronidase activity was observed in all nine ant venoms tested by Schmidt *et al.* [102], however, the activity was lower in comparison to that observed in wasp venoms. The ants with the highest hyaluronidase activity were the tropical ants *Paraponera clavata* and *Ectatomma tuberculatum* [102]. Other ants which have been found to contain hyaluronidase activity in the venom are *Myrmecia pyriformis* [104], *Pseudomyrmex triplarinus* [90] and *Solenopsis invicta* [86].

Proteases are responsible for moderate necrosis in some tissues of patients following envenomation by various venomous animals. Little information is available on venom proteases in insects, especially in ant venoms [82], and clinical reports of necrosis from ant stings are likely a result of secondary bacterial infections [60]. However, proteases have been reported in *Eciton burchellii* in very high levels [102]. Transcriptomic analysis of the venom gland of the ant *Tetramorium bicarinatum* suggested that the main toxin-like proteins are metalloproteinases that degrade proteins and hydrolyse specific peptide bonds [85]. The presence of a metalloproteinase in ant venom is significant as they are thought to be involved in disruption of the host′s coagulation cascade as well as in generating a more digestible prey [85]. A metalloproteinase has also been found in the venom of the fire ant *Solenopsis invicta* using proteomics techniques [83]. In wasps, metalloproteinases have been associated with inflammation, necrosis, oedema, and skin damage after massive attacks on humans [83].

Other proteases which have been identified in several hymenopterans and some ants are carboxylesterases [69,105]. These enzymes hydrolyse carboxylic acid esters into acids and alcohols; this enzyme has been considered to have a protective function for the organism as it promotes cellular detoxification by inactivating carcinogens and toxicants. Pesticides and drugs usually contain ester moieties that are susceptible to these enzymes and are therefore degraded by this enzyme. This enzyme has been found in the genomes of the ants *Harpegnathos saltator*, *Camponotus floridanus*, *Acromyrmex echinatior* [106], and, more recently, in *Dinoponera quadriceps* through transcriptomic analysis [69].

Another interesting finding of the investigation by Schmidt *et al.* [102] was the presence of phosphodiesterase activity in the venom of the ants *Ectatomma tuberculatum* and *Paraponera clavata*. Phosphodiesterases are more common in snake venoms and have not yet been reported in insect venoms [102,107]. They have been associated with catalysing the action of other active or toxic venom component functions [102] which can cause cell lysis or DNA/RNA degradation in the prey [107].

Several enzymes potentially involved with targeting major host defence cascades were also revealed through transcriptomics analysis of *Tetramorium bicarinatum* venom [85,91]. An example of such a protein is phenoloxidase which is a multicopperoxidase which generates highly reactive and toxic quinine intermediates that clear bacterial infections from the insect. This is because they cross-link bacteria to a protein on the cytoplasmic membrane of haemocytes [108,109]. This protein has been identified as an important venom compound among parasitic wasps to disrupt their host′s immune systems.

#### 4.1.3. Proteins that Promote Tissue Damage or Cause Inflammation

Phospholipases are a common protein found in ant and other hymenopteran venoms that, in addition to the activities described in Section 4.1.1, cause disruption of the phospholipid membrane leading to pore formation, inflammation, and cell lysis [83,110,111]. One of the earliest reports of phospholipase activity in ant venoms was in the bulldog ant *Myrmecia pyriformis* [112]. However, since then many other ants have been reported to express phospholipases in their venom either through enzymatic, proteomic or transcriptomic studies. For example, the ants *Paraponera clavata, Pogonomyrmex occidentalis, Pogonomyrmex badius, Eciton burchellii* [102], *Pseudomyrmex triplarinus* [90], *Dinoponera grandis, Solenopsis invicta*, and *Ectatomma tuberculatum* [96] have all been reported to have phospholipase activity using enzymatic studies. Through transcriptomic techniques, the additional ant species *Dinoponera quadriceps* [69] and *Tetramorium bicarinatum* [91] have also been reported to have phospholipases. According to current data, the phospholipase activity of ant venoms seems to be lower than that of wasps. While the activity of *Pogonomyrmex badius* is comparable to that of the yellow jacket wasp [102], *Tetramorium caespitum* has with no reported phospholipase activity [89]. This indicates that venom phospholipase activity is not broadly distributed in all ant species.

As previously mentioned, PLDs have not been reported in hymenopteran venoms, however they have been recently predicted in two ant venoms, *Dinoponera quadriceps* and *Solenopsis invicta* [69,83]. The presence of PLD in ant venoms is a significant finding as it has only been previously reported in spider venoms, highlighting the need for confirmation by enzymatic assays. The enzyme has been often referred to as sphingomyelinase D where it can hydrolyze sphingomyelin containing membranes, or phospholipase D by virtue of its wider spectrum of lipid substrates. In the brown spider *Loxoceles gaucho,* sphingomyelinase activity results in characteristic dermonecrotic lesions, which typically follow a massive inflammatory response [113,114].

The 2D-PAGE analysis of *Solenopsis invicta* [83] revealed the presence of several other proteins that could promote tissue damage. These included myotoxin 2-like proteins previously found in snake venom proteins that have been reported to cause necrosis of tissue by increasing cytolysis and microvascular permeability [83,115] and PSTx 60-like protein previously identified from sea anemones which also promotes tissue damage due to a haemolytic action [116].

Venom acid phosphatases are common toxic inflammatory enzymes in venoms and have known digestive functions and toxic actions to cause histamine release and cell lysis [117,118]. Venom acid phosphatases also seem to be common in ant venoms in different levels of abundance, with *Eciton burchellii* and *Ectatomma tuberculatum* having the highest reported activity [98,102]. Interestingly, ants have higher activities of this enzyme overall compared to wasp venoms [102]. This enzyme is also a typical tissue enzyme, so it has been suggested that it might be a contamination from the venom gland tissues. However, venom collected by electrical stimulation was found to contain these proteins suggesting this is a true venom protein [102].

#### 4.1.4. Allergens

An allergen is any substance capable of eliciting an allergic reaction. Often, this can culminate in anaphylactic shock, which is a serious reaction involving oedema and systemic smooth muscle stimulation. The autacoid histamine is one of the key molecules involved in the mediation of hypersensitivity. Whilst all known allergens are relatively large molecules, with most allergens consisting of proteins or protein conjugates, the exact chemical properties leading to allergenicity are not well understood. There are also other substances which can induce the release of histamine, potentially leading to a hypersensitive state. In summary, (i) different families of proteins may work as potent allergens, although this cannot be reliably predicted from their sequence, nor does it depend on enzymatic activity; and (ii) some enzymes and non-proteinaceous substances can induce histamine release, either directly or by activating the immune system via local reaction products, thus also acting as allergens.

Allergic reactions, as well as anaphylaxis, are a common manifestation of stings by species of the order Hymenoptera, and ants are no exception. Indeed, it has been reported that perhaps over 50% of venom secretion proteins are allergenic proteins [85]. In Australia, most allergic reactions to ants are attributed to ants of the genus *Myrmecia*, particularly the jack jumper ant *Myrmecia pilosula* [43,74,80]. This ant has been alleged to rival the fire ant *Solenopsis invicta* in terms of venom allergenicity. Within hymenopterans, proteins ranging from 20 to 50 kDa are usually the source of these allergenic effects [80]. However, up until fairly recently, most allergenic activity was attributed to the peptide components. For example, *M. pilosula* allergenicity was mainly attributed to the pilosulins, however, seven proteins (20–90 kDa) have now been discovered and are believed to contribute to the allergic effects manifested after a sting [74,81].

**Figure 2:**
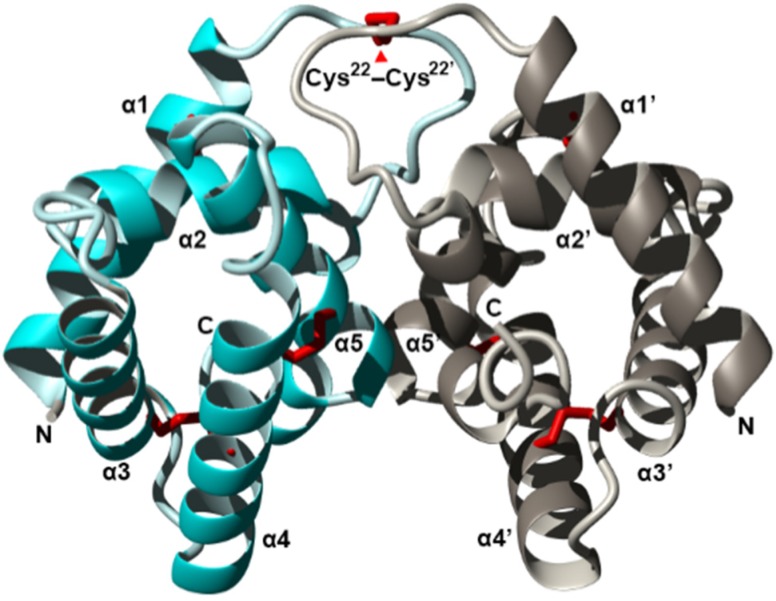
Crystal structure of the fire ant (*Solenopsis invicta*) venom allergen Sol i 2 dimer (PDB accession 2YGU) shown as a ribbon diagram. The two monomers (cyan and silver) dimerized by a disulfide-bond on symmetrical residues Cys^22^. Each monomer is composed of five α-helices, with helices α2–α5 surrounding a central hydrophobic cavity. Figure modified from Borer *et al.* Redrawn from [122].

**Figure 3:**
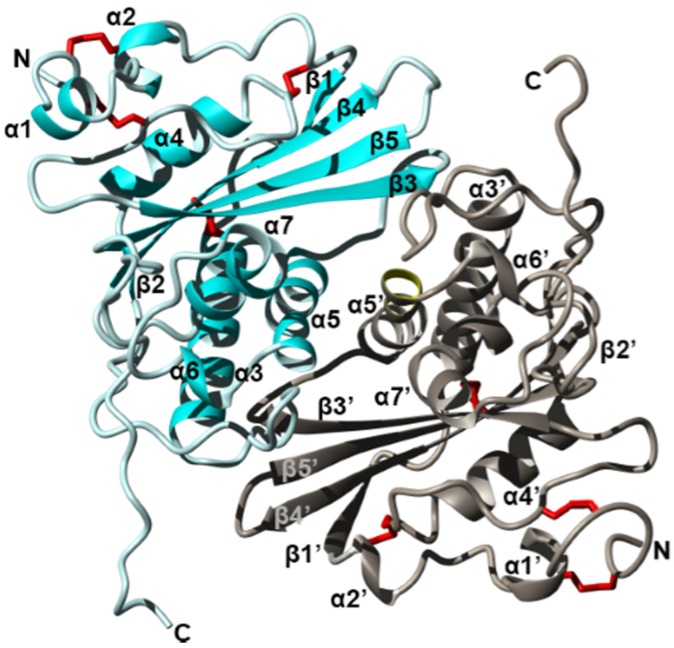
Crystal structure of the fire ant (*Solenopsis invicta*) venom allergen Sol i 3 dimer (PDB accession 2VZN). Ribbon diagram revealing the overall structure of each monomer which contains seven helices (α1–α7) and five beta strands (β1–β5), arranged as three stacked layers, giving rise to an α–β–α sandwich. Two units (cyan and silver) form a dimer by non-disulfide bonds involving symmetrical residues in helix α5 and α5′. Disulfide bridges are shown in red, and N and C termini are labelled. Figure modified from Padavattan *et al.* Redrawn from [125].

An example of an ant whose allergenicity was attributed to proteins is that of *Solenopsis invicta* whose four main allergens (Sol i 1–4) are between 14 and 37 kDa [98,99,119,120,121]. Initially, it was believed that they all possessed phospholipase activities which was causing the allergenic effects [98], due to previous work suggesting that phospholipases cause the release of histamine [104]. A pioneering study on these allergens using enzymatic assays ruled out hyaluronidases and venom acid phosphatases as the cause of the allergenic effects of fire ant venoms [98]. To date, little is known about the biological activities of most of these proteins. Sol i 1 contains both phospholipase A_1_ and B activity and was shown to be more related to vespid phospholipases than bee phospholipases [83,99]. Sol i 2 has had its crystal structure recently determined (see Figure 2) [119,122], and was suggested to play a role in binding temporarily to hydrophobic factors such as trail pheromones [122]. Sol i 3 is a dimeric protein that is a member of the antigen 5 protein family with no known enzymatic activity (see Figure 3) [83,123,124,125]. Sol i 4 is homologous to Sol i 2 in sequence but occurs as a monomer [122] its biological function is also still unknown. Like Sol i 2, it is unique to ant venoms and does not seem to be homologous to any bee or vespid proteins [83,124]. Further potential allergens have been recently identified among fire ant venom proteins, which proved to be more diverse than previously thought, however specific immunological tests are necessary to confirm which ones are the most allergenic proteins [83]. Homologs of Sol i 2 and Sol i 3 have been predicted from the transcriptome of *Dinoponera quadriceps,* however, they have several unique amino acids which show species-specific diversification of these proteins [69].

*Brachyponera chinensis* (formerly *Pachycondyla chinensis*) is another ant whose proteins account for the majority of the allergic manifestations. Thus far, nine proteins (all >10 kDa) have been identified as allergenic using 2D-PAGE and western blots. The major allergenic protein seems to be a 23 kDa protein with a p*I* 8.7 given that the majority of IgE proteins from hypersensitive patients reacted against this protein. This protein was found to be homologous to *Solenopsis invicta* Sol i 3 and the antigen 5 family of proteins [124].

#### 4.1.5. Antimicrobial Proteins

Antimicrobial proteins have bactericidal activity and include proteins with a colony asepsis role, preventing contamination of stored food as well as colony individuals, including the brood. An interesting group of proteins involved with colony asepsis from *Solenopsis invicta* venom are the bactericidal transferrins [83]. These proteins chelate free Fe^3+^ in biological fluids, making it unavailable for use by bacteria that need it for their survival [126].

### 4.2. Identified Proteins with Unknown Functions

Due to the lack of proteomic data, a recurring issue with ant venom proteomic studies is the abundance of unassigned and unannotated predicted proteins in database searches. According to published venom gland transcriptomes [69,85,91], there are thousands of unique hypothetical proteins which could not be assigned to any biological function or previously described protein through sequence searches. Such proteins could include unique venom toxins that could be investigated as potential insecticidal or antimicrobial drugs.

## 5. Ant Alkaloids

Alkaloids are defined as a heterogeneous assembly of secondary metabolite cyclic compounds containing nitrogen atoms in a negative oxidation state [127]. Nowadays, around 14,000 different alkaloids are known [127] with the inevitable result that the chemistry of alkaloids is very complex with numerous chemical subdivisions. They are primarily found in plants, particularly in the Angiosperma, where alkaloid production pathways seem to have diversified mainly as a protection against defoliation by herbivores [128]. However, it is not only plants that contain alkaloids, with a number of alkaloids having been isolated from fungi, and different classes of vertebrates (e.g., numerous toads, the musk deer, and beavers) and invertebrates (mainly marine sponges, myriapods, and insects). Alkaloid-rich insects are particularly prevalent among lepidopterans, beetles, and ants [129].

Alkaloids in ants were first reported in the early 1970s [130] and they have been reported in an increasing number of different ant groups, particularly as venom secretions (for a summary see Table 4). Although wasp venoms may contain amines and several other low molecular weight compounds, venom alkaloids seem to be a particularity of ants within Hymenopterans [127,131].

**Table 4:** Ant genera containing venom alkaloids.

Subfamily	Ant Genus	Structural Family	Trivial Name	Reference
Myrmicinae	*Atta Acromyrmex*	Pyrroles	Trail pheromone	[132]
	*Messor*	Pyridines	Anabaseine Anabasine	[133,134]
	*Aphaenogaster*	Pyridines	Anabaseine	[135]
	*Megalomyrmex*	Pyrrolidines Pyrrolines Pyrrolizidines	-	[136]
	*Monomorium*	Farnesylamine Pyrrolidines Indolizidines	Monomorines (trail pheromones)	[137]
	*Myrmicaria*	Polycyclic indolizidines Pyrrolo-indolizidines	Myrmicarins	[138]
	*Solenopsis*	Piperidine and piperideine Dialkylpyrrolidines and Pirrolines Indolizidines	Solenopsins Histrionicotoxins Gephyrotoxin	[139,140,141]
	*Carebarella* ^1^	Pyrrolidines	Histrionicotoxins Gephyrotoxin	[142]
	*Leptothorax* *Harpagoxenus*	Alkylpyrrolidines	–	[143]
Formicinae	*Nylanderia* *Brachymyrmex*	Alkyl-hydroxyl-indolizidines	Pumiliotoxins ^2^	[144]
Pseudomyrmecinae	*Tetraponera*	Pyrimidines	Tetraponerines	[145]

^1^ This genus has been very recently incorporated within *Solenopsis* [146]; ^2^ Pumiliotoxins are important alkaloids isolated from mixed whole ant extracts of other groups, but as yet it is unknown whether these alkaloids come from the venom apparatus.

**Figure 4:**
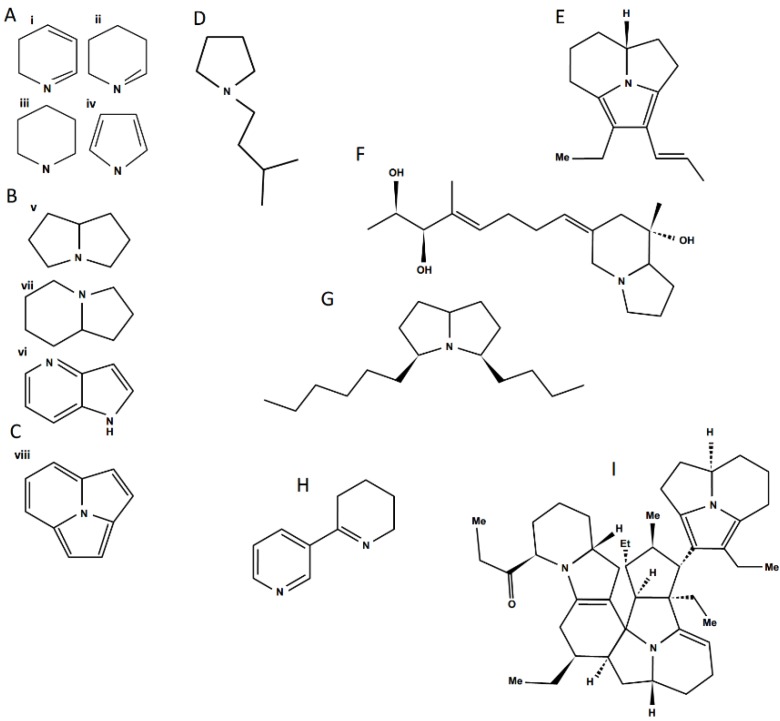
Structural diversity among ant venom alkaloids. A—monocyclic alkaloid families, i. Pyridine, ii. Piperideine, iii. Piperidine, iv. Pyrrole; B—bicyclic alkaloid families, v. Pyrrolizidine, vi. Indolizidine, vii. Pyrrolopyridine; C—trycliclic alkaloid family, viii. Pyrroloindolizidine; Examples of ant alkaloids: D—anabaseine; E—pumiliotoxin; F—monomorine; G—another *Monomorium* venom alkaloid; H—anabaseine; I—a complex myrmicarin.

**Figure 5:**
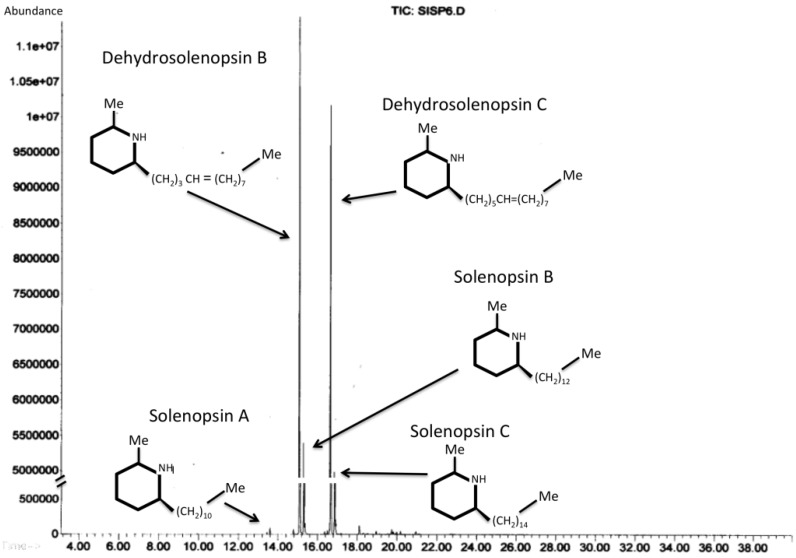
Examples of the most abundant venom alkaloids found in the venom of *Solenopsis invicta*. GC-MS chromatogram of hexane solvent in which fire ant workers were immersed.

In the following sections, only alkaloids with toxic activities found in ant venoms are discussed; for an overview of general alkaloid structures and classification see Anisewiski [127]. Ant alkaloids can be either monocyclic, bicyclic, tricyclic or polycyclic (the latter being derived from tricyclic alkaloids), thus displaying considerable diversity (Figure 4). The venom from ants of the same species group may contain several different alkaloids and isomers, however they all tend to share the same basic structure (exemplified by the alkaloids from the venom of fire ant workers in Figure 5). Venom alkaloids in ants, particularly in those groups where they are predominant compounds, play a central role in their biology.

### 5.1. Production of Alkaloids in Ant Venoms

As previously mentioned, most alkaloids are primarily described from plant extracts, illustrated by the well-studied compounds coniine and nicotine. Plants take advantage of secondary metabolites, such as alkaloids, as a deterrent against herbivores. Therefore, it is often presumed that ants can sequester alkaloids into their venoms by feeding on plants [147], however, laboratory ant colonies can produce alkaloids in the absence of such foods, thus demonstrating they synthesize the compounds directly (personal observation by EGPF). This observation singles out ants, from other hymenopterans, for their capacity to produce copious amounts of bioactive alkaloids, with different groups of ants producing particular groups of alkaloids (Table 4). Venom alkaloids are believed to be produced inside the convoluted gland of the venom apparatus, as mentioned in anatomical studies of the venom apparatus of *Solenopsis* fire ants [148,149]. These authors even demonstrated local tissue damage possibly caused by synthesis and storage of such toxic compounds. Exactly how these ants produce the alkaloids is presently unknown, however, a few biochemical pathways have been proposed [150]. Interestingly it has been mentioned that among the transcripts of a fire ant venom gland, there are several enzymes related to the mevalonate pathway of synthesis of polyketides, which is the biochemical pathway attributed to this class of alkaloids [20]. It is also possible that a microbial symbiont may have been involved in the production of intermediary compounds, as was suggested for some alkaloids found in sponges [151] and briefly hinted at in ants by Saporito *et al.* [144].

To date, alkaloids are known to be prominent within the venom secretions of the subfamily Myrmicinae, particularly within the tribe Solenopsidini, which includes such genera as *Solenopsis*, *Monomorium*, *Allomerus*, and *Megalomyrmex*. These ants usually either infest the nests of other species (e.g., thief ants within *Solenopsis* and *Megalomyrmex*) or they are slow-moving hardy foragers such as the flower ants from the genus *Monomorium*. Alkaloids are usually bitter and frequently poisonous when ingested, thus these ants are granted protection against most potential predators. Moreover, venom alkaloids aid these ants to manipulate and avoid their host species and competitors.

The literature concerning the biological activities of alkaloidal compounds detected in ant venoms is fragmented. Ant alkaloids have only been investigated in a minority of ant species where they are associated with a range of biological activities, with different activities often reported for the same compounds across different ant groups. Nevertheless, several alkaloids detected in ants are shared with other distant organisms (e.g., anabaseine alkaloids are also found in nemertine worms, and tobacco plants), and a few have been synthesized for specific investigations (e.g., synthetic solenopsins for biomedical studies). In such instances, there are studies of their chemical and biological properties published in sources unrelated to myrmecology (see Anisewiski [127]). Non-toxic activities include those relating to communication and behavioral modulation including trail, alarm and sex pheromones and attractants [131,152] but these are outside of the scope of this review and will not be covered here. For a more complete overview on the biological activities of ant venom alkaloids, please refer to Jones & Blum 1983, Brossi 1987, Escoubas & Blum 1990, Anisewisky 2015 [127,153,154,155].

Alkaloids with toxic adverse effects on other organisms include: (i) herbicidal effects recorded from the alkaloid-rich venom of *Solenopsis* [153]; (ii) arthropod toxins that are used against competitors, predators, and prey either by spraying, injection or topical application, mainly recorded from *Monomorium*, *Solenopsis*, and *Tetraponera*; (iii) antimicrobials which remain to date poorly studied in ant venoms excepting studies with *Solenopsis* and some tests with *Monomorium*; and (iv) mammalian toxins, as demonstrated by tests on mammals and mammalian cells, reported mainly from *Solenopsis*, but also from *Monomorium* and *Tetraponera* [156]. Considering the disproportionate number of studies regarding the toxic effects of fire ant venom alkaloids, they are discussed in further detail below.

### 5.2. Solenopsins: A Case Study of Ant Venom Alkaloids

The chemistry and physiological effects of venom alkaloids have been best studied among the fire ants (*Solenopsis* spp.; Myrmicinae), a notable group of about 20 species [157]. Compared to other *Solenopsis* ants which typically behave as thief-ants, fire ants are much larger and faster, foraging in the open and using venom to subdue larger prey, defend their ground and food, and discourage predators. They are widely reputed for their aggressiveness combined with the burning sensation caused by their stings [157]. When injected into the skin their poorly-soluble venom alkaloids cause a local inflammatory reaction, and can lead to pustule formation within hours. Due to some fire ants being regarded as one of the top-rated global invasive pests, there is a growing body of literature about their biology and venom alkaloids.

#### 5.2.1. Solenopsin Chemistry

Alkaloids in fire ant venoms are mainly hydrophobic piperidines called solenopsins (generally similar in structure to coniine and nicotine [127]) and piperideines in much lesser amounts. These are oxygen-free polyketide alkaloids as their nitrogen atom is inserted into a polyketide carbon skeleton, and they are not metabolically derived from amino acids [127]. Structurally, these are compounds with a piperidinic ring, often unsaturated, attached to the side by a hydrocarbon chain of variable length [127] (Figure 2). The solenopsins come in many isomeric forms with slightly different chemical and biological properties for each configuration [158]. There are several piperideines found in trace amounts in the venom, which are currently thought to be unstable intermediates for the synthesis of solenopsins, but these remain largely unstudied [158].

The solenopsins can be easily extracted from *Solenopsis* ants either by directly dissecting the venom glands or by dipping the ants in organic solvents [21,159]. The extract can then be partially purified through traditional thin-layer or silica column chromatography based on the relative affinity of solenopsins for silicates and different solvents. Given this facile extraction procedure, copious amounts of venom can be obtained from whole nests (further details are given in Fox *et al.* [84]). Female individuals of any given caste will carry a unique mixture of solenopsins [130], as shown in Figure 3. Unfortunately, because of the shared chemical properties between the different isomers, complete purification of each of these compounds is currently not feasible [160]. Thus, until preparative purification methods to separate isomers are devised, the study of the biological and physiological effects of solenopsins depends either on the synthesis of each compound, or testing with natural mixed extracts (see Fox [20]).

#### 5.2.2. Solenopsin Pharmacology

In general, solenopsins are regarded as the main toxic component of fire ant venoms. Apart from a burning sensation, oedema and pustule formation, they have been found to possess necrotic, haemolytic, antibiotic, and insecticidal activities [23,153]. Many alkaloids have antimicrobial properties to prevent infections from materials and prey brought into the colony. Solenopsins, in general, are no exception with potent antimicrobial activity against fungi and gram-positive bacteria [161,162], while solenopsin A was effective against gram-negative bacteria. The ants appear to employ this activity to disinfect their surroundings and brood by vigorously shaking their gaster and spreading the venom throughout the nest [163,164]. Also, solenopsins are effective as insect repellents and as insecticides, mainly against lepidopterans [165,166]. Such a property is invaluable to these ants since they are aggressive predators and competitors of other ants compared to thief ants which invade the nests of other ants to pillage resources and the brood.

In mammals, solenopsins were demonstrated to cause a number of complex physiological alterations, such as blockade of the neuromuscular junction [167], triggering histamine production in mastocytes [168], inhibiting ATP-dependent sodium-potassium pumps, and respiratory chains [169,170], activating platelets and neutrophils [171], and inhibiting neuronal nitric oxide synthase [172]. Following intravenous injection, synthetic isosolenopsin A and solenopsin A were capable of severely impairing both the central nervous system and cardiovascular systems of mice [173], which shows their capacity to cross the blood-brain barrier. Doses of 3–30 mg/kg were particularly toxic, causing a range of effects from dizziness, cardiorespiratory complications, seizures, and death [173]. These toxic effects are beneficial to these ants as active predators of both vertebrate and invertebrate prey, and also in defending their nests. Synthetic solenopsin A has been shown to possess a potent inhibitory activity against class-1 phosphatidylinositol-3-kinase signalling and angiogenesis in mice embryos and zebra fish, making this alkaloid a potential lead therapeutic for the treatment of cancer [174].

## 6. Other Toxins

A set of additional small molecule compounds have been found in ant venoms. This includes alkylated pyrazines that are usually not considered alkaloids [127], being more common as mandibular gland secretions, identified as venom components in some ants (e.g., *Atta bisphaerica*) [152,175]. The venom glands of some species have also been reported to contain monoterpene hydrocarbons. For example, the primary venom compound of *Myrmicaria natalensis* is the cyclic terpene limonene [176], however, the venom also contains α-pinene, β-pinene, sabinene, terpinolene, β-myrcene, α-phallendrene, α-terpinene, and caphene [177]. All of these compounds can be highly toxic to insects minding the fact that this ant species preys on termites. The presence of immunologically active heterogeneous polyanonic polysaccharides have also been reported in the venoms of *Pseudomyrmex* spp. [178]. There are also non-alkaloidal amines such as pteridines that have been identified from some ants including *Formica* and *Lasius*, actidines found in *Megaponera* and *Dorymyrmex*, and histamine which is abundant in venoms of *Myrmecia* spp. [131,179].

Finally, it should be mentioned that the secreted venom of ants is even more complex due to interactions with secretions from their Dufour′s gland. The Dufour′s gland is an accessory organ attached to the venom gland and considered part of the venom apparatus [180]. Its secretions may act synergistically with toxins originating from the venom gland and contribute to the toxicity of the secreted venom. For example, the Dufour′s gland of *Crematogaster scutellaris* produces long-chain primary acetates (non-toxic) which are converted to highly electrophilic aldehydes (toxic) by enzymes from the venom gland during the venom secretion [181].

## 7. Conclusions

A host of recent studies have revealed that ant venoms are more complex and heterogeneous than initially thought, owing in particular to the newly uncovered complexity of their peptidome and proteome contents. Although the extant biodiversity of ant venoms remains largely unexplored, their high plasticity is suggested by recent studies of a variety of subfamilies or genera, showing highly different venom compositions. Formicinae ants and some Myrmicinae genera essentially produce non-proteinaceous venoms primarily composed of formic acid and alkaloids, respectively. In contrast, most ant species from other subfamilies have retained the ability to sting and, in turn, produce peptide- and protein-rich venoms. Evolution has therefore led to some ants abandoning their ability to sting, unlike wasps and most Apidae, whilst evolving a different set of chemical defenses along with modified predatory and defensive behaviours. To date, the molecular and structural complexity of ant venoms has barely been explored but the broad ecological diversity of ants strongly suggests that further structural peptide and protein diversity might be uncovered by extensive biochemical studies, leading to discovery of a much broader range of toxins than currently observed. Technological progress, particularly in deep-sequencing approaches, coupled with high-end transcriptomic, peptidomic, and enzymatic methods based on mass spectrometry and peptide *de novo* sequencing, will quickly allow for the detection and characterization of numerous novel peptides and enzymes, at sensitivity levels and a depth not previously attained. Despite the biochemical diversity potentially present in ant venoms, this review highlights the paucity of knowledge on the molecular pharmacology of most ant toxins. The biological function and mechanism of action of the majority of ant venom toxins described to date remain poorly characterized or simply unstudied. Thorough exploration of a broader taxonomic diversity will likely result in the discovery of novel bioactive toxins which may become useful tools for biopesticide or drug development and will shed insights on the molecular evolution of venom in Hymenoptera, the roles of venom in ant biology, the genetic makeup leading to ant venom diversification and its role in the successful conquest of almost all ecological niches by ants. The characterization of functional roles and pharmacological properties of this vast array of novel toxins (particularly peptides) will certainly become one of the most functional and significant endeavors in future ant venom research, with a high application potential. Thus, the whole world of ant venom is still a vast uncharted scientific territory awaiting our attention.
